# Enhancement of Lipid Stability and Acceptability of Canned Seafood by Addition of Natural Antioxidant Compounds to the Packing Medium—A Review

**DOI:** 10.3390/antiox12020245

**Published:** 2023-01-21

**Authors:** Santiago P. Aubourg

**Affiliations:** Marine Research Institute, Spanish National Research Council (CSIC), c/E. Cabello, 6, 36208 Vigo, Spain; saubourg@iim.csic.es

**Keywords:** canning, seafood, packing, oils, plants, algae, by-products, lipid oxidation, ω3 fatty acids, quality

## Abstract

Seafoods are known to include high contents of valuable constituents. However, they are reported to be highly perishable products, whose quality rapidly declines post-mortem, thus demanding efficient processing and storage. Among the traditional technologies, canning represents one of the most important means of marine species preservation. However, owing to the thermal sensitivity of the chemical constituents of marine species, remarkable degradative mechanisms can be produced and lead to important quality losses. The demand for better quality food makes the need for advanced preservation techniques a topic to be addressed continually in the case of seafood. One such strategy is the employment of preservative compounds obtained from natural resources. The current review provides an overview of the research carried out concerning the effect of the addition of bioactive compounds to the packing medium on the thermal stability of canned seafood. This review addresses the preservative effect of polyphenol-rich oils (i.e., extra virgin olive oil) and different kinds of products or extracts obtained from plants, algae and seafood by-products. In agreement with the great incidence of lipid damage on the nutritional and acceptability values during high-temperature seafood processing, this work is especially focussed on the inhibitory effect of lipid oxidation development.

## 1. Introduction

Seafoods are reported to provide great proportions of valuable constituents for the human diet such as highly unsaturated fatty acids, nutritional proteins, lipid soluble vitamins (namely A and D) and macro- and microelements (I, F, Ca, Cu, Zn, Fe, Se and others) [1]. Among such constituents, the lipid fraction has attracted great attention due to its high content of ω-3 polyunsaturated fatty acids (PUFA). On the basis of recent research, this fatty acid group has shown a positive role in preventing different kinds of human diseases [2,3].

Marine fish and invertebrates constitute highly perishable products whose quality rapidly declines during processing and storage. It has to be taken into account that such food products are obtained from poikilothermic organisms with both a soft muscular and skin structure, a high water and non-protein nitrogen content and a low collagen content [4]. Furthermore, the highly unsaturated lipid composition is shown to be especially prone to the development of lipid oxidation. As a consequence, such products are among the most perishable foods, and require a rapid and efficient treatment during the different processing and storage steps.

Lipid oxidation compounds have shown a great importance for the food industry. In addition to the possible effects on human health in the form of oxidative stress or oxidative damage [5], there are major concerns in food technology resulting from lipid oxidation due to the formation of oxidation products such as fatty acid hydroperoxides and carbonyl compounds (i.e., primary and secondary lipid oxidation compounds) [6]. As a result, off-flavours with characteristic rancid odours are produced and can be responsible for the decrease in both the nutritional quality and safety of seafood.

In order to prevent the development of lipid oxidation in food in general, synthetic antioxidants have been used abundantly to retard or minimise oxidative deterioration of foods, such as butylated hydroxyanisole (BHA), tertiary butyl hydroquinone (TBHQ) and butylated hydroxytoluene (BHT) [7]. However, consumers are more and more concerned with synthetic antioxidants because of their carcinogenicity. The use of naturally occurring antioxidants nowadays has been regarded as an effective way to promote human health, taking into account their preservative effect on seafood and food in general [8,9].

Among the different kinds of traditional technologies, canning represents an important strategy for seafood preservation [10,11]. According to recent FAO statistics (2010–2019), about 9.5–11.5% (ca. 16.3–17.5 million tonnes per year) of total world fishery production corresponds to marine species canning [12]. Canned seafoods are produced from a variety of raw materials, including eviscerated small fish, fish muscle mass in whole slices, chunks, fillets, flakes or in shredded form, or molluscs, with or without a shell and include diverse culinary preparations such as salads or complete meals. In this technological treatment, marine species are introduced in sealed hermetic cans with different kinds of packing media (oil, brine, pickle, etc.) followed by a sterilisation procedure, in which the canned muscle is heated at a temperature range of 110–130 °C for a period of time of 25–120 min [13]. The canning process allows the market to distribute seafood on a global scale, taking advantage of a broad time frame for distribution, storage and consumption under strict food safety conditions.

Heat processing alters the general aspect and flavour of the starting material so that a very different final product is obtained [14]. This is achieved with the utilisation of a high temperature for an extended period, so that both the enzymes and bacteria should be permanently inactivated by heat and, provided that recontamination does not occur and no negative interaction with the container is produced, heat-processed seafoods are preserved for a very long time [15]. The duration and temperature during the heating process and the required cooling cycle must be properly assessed. Long-term high temperature–high energy consumption increases the seafood product cost and causes the loss of sensory quality such as soft texture, the separation of jelly and fat, discolouration and undesirable heat treatment taste. Additionally, owing to the thermal sensitivity of a large number of chemical constituents, several degradative pathways can be observed such as the oxidation of vitamins and lipids, heat degradation of the nutrients in general, leaching of water-soluble vitamins, minerals and proteins into the packing medium, toughening and drying of fragile marine protein and the development of non-enzymatic browning [16,17].

In connection with an increasing trend of seafood consumption, public health concerns have become an issue requiring careful attention, not only to ensure quality and nutrition, but also safety, as the major challenges faced by the food trade and technologists. Consequently, the demand for better quality processed food, together with the high demand far from local fishing ports, drives the need for advanced preservation techniques to be addressed continually in the case of seafood. Concerning canned seafood, different advanced strategies have been tested to enhance the quality of the product that the consumer receives. One such strategy is the addition of preserving compounds into the packing medium employed. Since great concerns have been reported for the employment of synthetic preservatives, additive compounds obtained from natural sources are perceived by consumers as safer and widely accepted.

The main objective of this review is to provide an overview of the research carried out concerning the effect of the addition of natural preservative compounds to the packing medium on the thermal stability of canned seafood. In agreement with the great incidence of lipid damage on the nutritional and acceptability values during the high-temperature processing of seafood, the current study particularly addresses the inhibitory effect on the development of lipid oxidation during the canning process. In order to carry out the corresponding bibliographic research, the Food Science and Technology Abstracts (FSTA) and the Aquatic Sciences and Fisheries Abstracts (ASFA) databases were consulted.

## 2. Effect of Packing Oil Composition on the Thermal Stability of Canned Seafood

### 2.1. General Aspects of the Preserving Properties of Packing Oils

Previous research has dealt with the effect of the oil-packing medium on the composition of the canned muscle of marine species. An important interchange of lipid material has been described between the oil-packing medium and the lipid fraction of the canned seafood muscle [18,19,20]. As a result, marked increases in the canned flesh have been detected for the content of fatty acids that are abundant in the packing oil employed (i.e., oleic and linoleic acids), while a decreased presence of highly unsaturated fatty acids (i.e., eicosapentaenoic acid, EPA, and docosahexaenoic acid, DHA) has been detected in canned seafood flesh.

The preservative effect of the packing oil on the lipid damage in the seafood muscle has been attributed to the presence of natural antioxidants present in the packing oils. Among such packing oils, polyphenol-rich olive oil (OO), a key component of the Mediterranean diet (MD), has acquired a great relevance. Obtained from olive fruits exclusively by physical–mechanical technologies (olive crushing, centrifugation, filtration, etc.), extra virgin olive oil (EVOO) is considered as a ‘lipid fruit juice’ [21]. The health benefits of OO in general are well known (i.e., nutraceutical and nutritional values); virgin OO (VOO) and EVOO have been pointed out among the main reasons that explain the health benefits attributed to the MD [22]. This effect has been especially important in populations with higher adherence to the MD, so that a marked reduction in the incidence of oxidative- and inflammatory-related pathologies, such as cardiovascular diseases, cancer and neurodegenerative disorders, have been detected [23].

Polyphenol-rich OO includes a wide range of constituents that are positively related to the prevention of lipid degradation during the different technological processing steps of marine fish and invertebrate species. The natural polyphenols present in both VOO and EVOO, especially in the latter, can act as free radical acceptors as well as metal chelators [24]. Remarkably, the shelf-life of the polyphenol-rich OO has shown to be higher than that of other vegetable oils; this advantage has been explained on the basis of the presence of phenolic molecules having a catechol group, such as hydroxytyrosol and its secoiridoid derivatives [25].

### 2.2. Research Performed on the Preservative Effect of the Packing Oil Composition

The effect of the packing medium on lipid hydrolysis development during the industrial canning of albacore tuna (*Thunnus alalunga*) was analysed using ^13^C-NMR spectroscopy [26]. In both media studied, brine and soybean oil, increases in the free fatty acid level were observed with preferential hydrolysis of PUFA esterified in the *sn-2* position of the glycerol moiety. It could be observed that the extent and mechanism of lipolysis were independent of the packing medium employed.

A comparative quality analysis was carried out by Lazos [27] by packing fresh water nase (*Chondrostoma nasus*) with 2% brine or olive oil. Among the findings, lower thiobarbituric acid reactive substance (TBARS) values were obtained in the canned fish corresponding to the oil-packing condition. In contrast, a higher formation of total volatile amines was detected in the fish packed in oil. No effect of the packing medium on the fish colour and appearance was detected.

The effects of different packing media (EVOO, refined OO, refined soybean oil and brine), differing in the content of natural antioxidants, were comparatively studied during albacore tuna (*T. alalunga*) canning [28]. Different rates of oxidation were observed among the four media after the thermal processing and storage of the tuna cans; thus, the EVOO showed potential antioxidant activity on the fish lipids. This ability was attributed to the solubilisation of hydrophilic phenols into the water–muscle interface. The phenolic composition of the EVOO showed a marked change after fish processing, suggesting phenol breakdown and strong interactions between the oil phenols and fish muscle components. On the other hand, the aqueous environment of the brine packing made the fish lipids more prone to oxidation development, presumably due to the accumulation of unsaturated fatty acids at the oil–water interface.

The polyphenols extracted from the EVOO were tested for their ability to inhibit lipid oxidation in canned tuna (*T. alalunga*) [29]. An antioxidant effect was observed in treated tuna by employing 400 ppm of the EVOO polyphenols in the packing medium, thus showing a similar effect as compared to 100 ppm of a 1:1 mixture of the synthetic antioxidants BHT and BHA. However, at concentrations lower than 100 ppm, the EVOO phenolic compounds promoted hydroperoxide formation and subsequent breakdown. The EVOO polyphenols were shown to be effective antioxidants when added to heated tuna muscle in the presence of either brine or refined OO. The EVOO polyphenols had higher antioxidant activity in the brine samples than in their counterparts canned with refined OO. As in the previous study, this higher antioxidant activity was explained by the authors on the basis of the greater affinity of unsaturated fatty acids towards the more polar interface between water and fish oil.

In order to investigate the antioxidant mechanism developed by the use of EVOO as a packing medium during fish canning, sealed cans packed with oil–brine mixtures (5:1, *v*/*v*) were developed [30]. The content of hydroxytyrosol, tyrosol, a combination of both and other phenols (i.e., dialdehydic form of decarboxymethyl oleuropein and decarboxymethyl ligstroside aglycons, and oleuropein aglycon) were shown to decrease in the oil phase after sterilisation with a marked partitioning towards the brine phase. The increase in total hydroxytyrosol and tyrosol content after processing, and the presence of elenolic acid in the brine, revealed hydrolysis of the ester bond of the hydrolysable phenols during thermal processing. It was concluded that both partitioning towards the water phase and the hydrolysis of phenols would contribute to the phenol loss from the EVOO in canned foods, as well as to the protection of ω3 PUFA in EVOO-canned fish products.

The oxidative and hydrolytic stability of the vegetable oils used as a liquid medium in canned fish was evaluated by Caponio et al. [31]. Sixteen canned tuna samples were tested, including two in EVOO, nine in OO (refined OO plus VOO) and five in refined seed oil (soybean, corn or sunflower oils). As a result of the interaction with the canned fish muscle, the fatty acid composition of the packing oils showed the presence of highly unsaturated fatty acids. *Trans* isomers were absent in the EVOO; in contrast, they were present in the OO and refined seed oil. On the basis of the formation of triacylglycerol oligopolymers, oxidised triacylglycerols and diacylglycerols (i.e., polar compounds), it was concluded that the use of EVOO as a packing medium marked an improvement in quality.

The effect of the fatty acid composition of the packing medium was comparatively studied by Tarley et al. [19] in sardine (*Sardinella brasiliensis*) canning in soybean oil and tomato sauce. The results indicated the detection of higher levels of EPA, DHA, total ω3, ω3/ω6 ratio and total saturated fatty acids (STFA) in sardines canned in tomato sauce. In contrast, sardines canned in soybean oil provided higher levels of linoleic and linolenic acids, total PUFA, total ω6 and PUFA/STFA ratio. No effect on the cholesterol content was detected, with values in the 50.4–65.1 mg/100 g range.

The influence of four different packing media (sunflower oil, soybean oil, OO and brine) on the lipid quality of canned silver carp (*Hypophthalmichthys molitrix*) was evaluated by Naseri et al. [32]. The hydrolytic rancidity showed that the free fatty acid contents in brine- and soybean-oil canned muscle were higher than in the canned samples corresponding to OO and sunflower oil packing. The highest conjugated diene values were found in the canned silver carp using brine as a packing medium. Except for the OO-canned muscle, substantial increases in TBARS values were obtained in all cases; the highest values being obtained in the soybean oil-canned samples. Tertiary lipid oxidation development, i.e., the interaction between primary and secondary lipid oxidation compounds and nucleophilic molecules present in the muscle [33], was measured by fluorescent compound formation. It could be observed that the samples canned in sunflower and soybean oil showed a higher fluorescent compound development than their counterparts corresponding to the OO and brine packing.

Changes in the fatty acid composition and lipid damage development in different packing oils (EVOO, OO and refined seed oil) present in different canned fish (tuna, sardine, anchovy and mackerel) were studied by Caponio et al. [34]. The results showed the lowest extent of both hydrolytic and oxidative degradation in the samples containing EVOO. The OO showed a higher hydrolytic degradation but a lower oxidative degradation and *trans* isomer content than refined seed oil. In most cases, the type of fish species did not influence the extent of the oxidative and hydrolytic degradation in the packing oils.

The effect of different packing media (sunflower, groundnut and coconut oils) on the sterilisation time required and on the quality of canned yellowfin tuna (*Thunnus albacares*) was assessed by Mohan et al. [35]. In this study, the lag phase for the sterilisation process was found to be lower for tuna packed in sunflower oil. A reduction in the total process time was observed in the tuna packed in sunflower oil, owing to a faster heating rate as compared to processing in coconut oil and groundnut oil. Additionally, heat processing in the sunflower oil medium showed a reduced rate of lipid oxidation development; this result was justified by its protective antioxidant activity. No effect on histamine content was proved as a result of the packing medium employed. The use of sunflower oil was recommended instead of coconut oil and groundnut oil due to its better heat penetration characteristics resulting in protective effects against lipid oxidation and hydrolysis.

Gómez-Limia et al. [36] studied the use of different packing media (sunflower oil, OO and spiced OO) on the oxidation parameters, antioxidant capacity and the total phenol and vitamin E contents in canned eel (*Anguilla anguilla*). After one year of storage, the sunflower oil and canned eel packed in this oil presented a higher antioxidant capacity and vitamin E content than OO, spiced OO or canned eel packed in these two oils. However, the total phenol contents were higher when OO or spiced OO were used as the packing media. These results were justified by the higher vitamin E value of sunflower oil and the higher content of polyphenols in OO.

The effects of the various steps of the canning process and of the different packing media (OO, corn oil, sunflower oil and high-oleic acid sunflower oil) on the vitamin contents in swordfish (*Xiphias gladius*) were studied by Cobas et al. [37]. It could be observed that the canning process caused the losses of some vitamins, particularly D3 and B9; however, the vitamin E content increased due to the preliminary frying step and the interaction with the packing oil. Canned swordfish packed in OO had lower vitamin A and E contents than the fish packed in sunflower oil. Swordfish packed in corn oil had the lowest vitamin A and B2 contents, while the samples packed in high-oleic acid sunflower oil had the lowest values of vitamins B9 and B12.

The effect of the packing medium (sunflower oil, OO or spicy olive oil) on the colour changes and sensory acceptance of European eel (*A. anguila*) was measured at different steps in the canning process [38]. The changes in colour parameters were shown to depend on the type of oil, the stage of the process and the canned storage time. The colour scores were higher in the canned eel packed in sunflower oil and spicy OO than in the canned eel packed in OO. Spicy OO was the packing medium in which the colour change was the greatest, probably due to the migration of some of the spice components into the oil. Concerning the sensory evaluation, the canned eel packed in sunflower oil was awarded the highest scores in consumer tests.

The effect of different packing media (water, brine, sunflower oil, refined OO and EVOO) on the fatty acid composition of canned Atlantic mackerel (*Scomber scombrus*) was analysed by Prego et al. [39]. In this study, the great presence of C18:2ω6 in the sunflower oil led to canned fish with high average PUFA and PUFA/STFA values and low average ω3/ω6 ratios when compared to canned mackerel corresponding to the other packing media considered (Table 1). However, the high presence of C18:1ω9 in both of the olive oils tested did not lead to remarkable increases in this fatty acid content in the corresponding canned fish product. The presence of total ω3 fatty acids, C20:5ω3 and C22:6ω3 did not provide differences in the canned fish muscle as a result of using different packing media.

## 3. Effect of Plant-Derived Compound Packing on the Thermal Stability of Canned Seafood

### 3.1. General Aspects of Preserving Properties of Plant-Derived Compounds

Since ancient times, spices and herbs have been added to food as seasoning additives due to their aromatic properties. Traditional and regional seafood processing are currently employed and provide valuable, safe and attractive products included in the human diet. Plant extracts are well known as bio-preservatives that have been shown to inhibit the microbial growth of Gram-positive and Gram-negative bacteria, yeasts and moulds and also exhibit useful antioxidant activity [40,41]. Among them, herbs of the Lamiaceae family, mainly oregano (*Origanum vulgare*), rosemary (*Rosmarinus officinalis*) and sage (*Salvia officinalis*), have been reported extensively as having remarkable preservative capacities. Although most of such plant products are classified as generally recognised as safe (GRAS), their use in food as preservatives can be limited because of flavour considerations, since the effective preservative doses may exceed sensorial acceptable levels [42].

In general, the total preservative capacity of fruit and vegetable extracts would reflect the concentration of a wide range of constituents such as ascorbic acid (vitamin C), alpha-tocopherol (vitamin E), beta-carotene (vitamin A precursor), various flavonoids and other phenolic compounds [43,44]. Among the main bioactive compounds identified in plant extracts, phenolic acids (e.g., p-coumaric acid, caffeic acid, rosmarinic acid and gallic acid), phenolic diterpenes (e.g., carnosic acid and epirosmanol) and flavonoids (e.g., aromatic compounds) can be mentioned [45,46].

Essential oils (also called ethereal or volatile oils) are aromatic oily liquids obtained from plant material by extraction, expression, fermentation, etc. Essential oils are known to scavenge free radicals and this property makes them important in health maintenance and disease protection [47]. Thus, they have shown antibacterial, antiviral, antimycotic, antitoxigenic, antiparasitic, insecticidal and antioxidant properties. The chemical composition of these oils depends on several factors (plant age, plant part, development stage, growing place, harvesting period, chemotype, etc.). Among such plant-derived compounds, phenolic volatiles like menthol (abundant in mint *Mentha canadensis*), carvacrol (abundant in oregano and rosemary), thymol (abundant in thyme *Thymus vulgaris*) and eugenol (abundant in clove *Syzygium aromaticum*) have been detected as the main bioactive ingredients [48,49].

### 3.2. Research Carried Out on the Preservative Effect of Plant-Derived Compounds as Packing Medium

Canned snakeskin gourami (*Trichogaster pectoralis*) products (pla-salid in tomato sauce, TS; chu-chee pla-salid in chilli paste, CC; tom-klong pla-salid in sour soup, TK) were prepared by Katuenggan et al. [50]. The effect of the processing conditions (sterilisation at 121 °C, *F_o_* = 12 min) on the product acceptability tested during storage was investigated. After a 2–3-month storage period, the consumer acceptance for the canned TS, TK and CC products were 88, 82 and 75%, respectively. According to the colour, odour, flavour, viscosity and texture assessment, the shelf-life time of all of the products was at least 3 months.

In a subsequent study [51], various snakeskin gourami (*T. pectoralis*) products (sauté pla-salid with garlic and pepper, KP; pla-salid chili dip, NP; fried rice with pla-salid, FR) were canned or packaged in retort pouches, sterilised and stored for up to 9 months. The shelf-life times of the KP, NC and FR products packed in cans and retort pouches were at least 6 months; during such period, all products showed a great sensory acceptability in all sensory descriptors (appearance, colour, odour, flavour and texture). After a 9-month storage, the consumer acceptance of canned products was 82, 93 and 67%, respectively.

Colembergue et al. [52] evaluated the chemical quality and the sensory acceptability of canned anchovy (*Engraulis anchoita*) preserved in tomato sauce. In their study, the initial fish was classified in three different batches according to body size. In all cases, the moisture, protein, fat and ash contents were around 74.2%, 9.3%, 3.8%, 2.3%, respectively; the energetic value of the anchovy being ca. 112.7 kcal/100 g. In the evaluation of sensory quality, the canned fish showed an acceptability rate ca. 89.9%.

Mohan et al. [53] analysed the effect of including different vegetable food (i.e., baby corn, green pea or broccoli) as packing ingredients during the brine (2% NaCl)-canning of yellowfin tuna (*T. albacares*). The thermal processing of tuna with an *F_0_* value of 8.0 min resulted in the reduction in process time of 4.35–15.22% when compared to canned tuna without vegetables in the same size cans. This reduced process time led to beneficial effects on the texture parameters (hardness, chewiness and springiness), lightness and sensory quality of tuna muscle. The sensory results indicated that the canned tuna with green peas or baby corn had better acceptability than their counterpart samples canned with broccoli.

Farmed cobia (*Rachycentron canadum*) was canned (121 °C for 40 min) by including Indian spice masala mix (a standard formulation including natural components such as onion, tomato, green chilies, coriander powder, red chili powder, cumin, garlic, fenugreek, mustard and vegetable oil) in the packing medium [54]. During storage at 30 ± 2 °C, the shelf-life time of the canned cobia was 12 months. Changes in the fatty acid composition revealed a relative increase in the canned muscle of C18:2ω6 and C18:3ω3 fatty acid contents, whereas those of C20:5ω3 and C22:6ω3 resulted in losses around 50%; a marked decrease in the ω6/ω3 ratio was also observed as a consequence of the canning process. The changes in the fatty acid profile of the fish muscle were explained as a result of the interchange with the fatty acids present in the vegetable oil.

With the aim of reducing the histamine content in different kinds of canned fish species (Pacific herring, *Clupea harengus pallasi*; Pacific pink salmon, *Oncorhynchus gorbuscha*; Pacific mackerel, *Scomber japonicus*) and ensuring its safety, cinnamon oil extract was added as a packing medium instead of soybean oil [55]. The cinnamon oil extract was obtained using the extraction of the powdered bark with soybean oil. The analysis showed that the histamine content in the fish samples canned with the cinnamon oil extract did not exceed the 35 mg · kg^−1^ level after 2 years of canned storage, while the control canned fish packed with soybean oil accumulated histamine up to 50 mg · kg^−1^. This preservative effect was explained on the basis of the presence of fat-soluble substances included in the cinnamon oil extract, showing an important inhibitory effect on enzymatic and microbial activity.

Demid et al. [56] addressed the development of a new delicacy of pasteurised canned fish. In this study, a semi-canned food was prepared from salmon (*Salmo salar*) fillets, pineapple fruit and OO. The optimisation of the process showed that the most acceptable composition of the canned product was obtained at a temperature–time mode of 85 °C for 60 min. Under such conditions, a high acceptability from the sensory panel was obtained (19.2 points on a 20.0-point scale). The microbiological stability, safety and high quality of the developed semi-canned food was proved during a 3-month storage period at 3.5 °C and at a 75% air humidity level.

Gomez-Limia et al. [57] evaluated the effects of the canning process, the packing medium (sunflower and OO), the spices (chilli and peppers) included as ingredients and the canned storage time (2 and 12 months) on the fatty acid profiles and lipid quality of European eel (*A. anguila*). The nutritional value (unsaturated fatty acid content) and the lipid quality increase (hypocholesterolemic and hypercholesterolemic fatty acid ratio and atherogenicity index) in the canned eel increased, owing to the absorption of the oil used in the canning process. The fatty acid profiles of the canned eel tended to be similar to those of the packing medium, so that an increase in the ω6/ω3 ratio was detected.

The processing technology of spicy canned commercial fish was studied by Li et al. [58]. The effects of the processing technology (previous marinating time, volume ratio of soy sauce to water and previous frying temperature and time) and the mass fraction of different seasonings (sugar, monosodium glutamate, pepper powder and chili powder) on the sensory quality of the canned fish were studied. After carrying out an optimisation process, the best results were reached by employing 0.2% or 9% of pepper powder, accompanied by 6 min of brine treatment, 1:5 of soy sauce:water ratio, 180 °C of frying temperature and 40 s of frying time, 4% of sugar, 0.5% of monosodium glutamate, 0.2% of pepper powder and 9% of pepper powder. The canned fish obtained using this method was golden in colour, chewy and spicy in taste.

The effect of a rosemary (*Rosmarinus officinalis*) extract and tomato juice on the lipid oxidation of sunflower oil-canned bonito (*Sarda sarda*) stored for 510 days at room temperature was studied by Kinay and Duyar [59]. An inhibition of TBARS formation was proved by the combined rosemary/tomato sauce treatment as well as a marked sensory quality retention. Lower pH values and a lower formation of total volatile amines were detected in the canned fish including rosemary extract as a packing medium.

The effect of grape seed (*Vitis vinifera*) and olive oil as a packing medium during sardine (*S. pilchardus*) canning was comparatively analysed by Bouriga et al. [60]. A lower TBARS formation was detected in sardines canned under the grape seed condition. This result was explained on the basis of the higher content of polyphenol compounds observed in grape seed oil than in olive oil. In both kinds of canned sardine, atherogenic and thrombogenic indices decreased after the canning process to less than 1.

Targueta Barreira et al. [61] evaluated the effect of including pink pepper (*Schinus terebinthifolius*) fruits during soybean oil-canning of sardines (*S. pilchardus*). A lower cholesterol oxide formation was detected as a result of the pink pepper addition. Chromatographic analysis (UHPLC/MS) showed the migration of antioxidant compounds (phenolic acids, flavonoids, tannins, etc.) from the pink pepper to the sardine muscle; this migration was found to be responsible for the cholesterol oxide decrease.

## 4. Effect of Alga-Derived Compound Packing on the Thermal Stability of Canned Seafood

### 4.1. General Aspects of Preserving Properties of Alga-Derived Compounds

Marine algae have been consumed in Asian countries (i.e., China, Japan and Korea) for centuries. An increasing interest is being detected in Western countries resulting from the search for new sustainable sources of healthy food and natural active products [62]. Both macroalgae and microalgae have shown to be a relevant source of beneficial constituents such as trace minerals, dietary fibre, lipids, vitamins and amino acids [63,64]. In spite of being exposed to a combination of high oxygen concentration and light, the lack of structural damage in algae organs has led to the consideration that their protection against damage arises from their content of preservative constituents [65].

Macroalgae, also known as seaweeds, are commonly classified as green (chlorophyte), brown (phaeophyte) and red (rhodophyte) algae. Recently, different kinds of industries (cosmetics, pharmaceutical, textile, fuel, plastics, paint, varnish and food) have carried out great efforts on the employment of macroalgae metabolites [66]. Related to food technology in general, seaweeds have attracted great attention because they contain a profitable variety of chemical components with potential antioxidant activity, this providing an interesting possibility to enhance seafood quality [67,68].

On the other hand, microalgae are photosynthetic microorganisms with a short cell-propagation cycle, easy large-scale culture, adaptability to various growth environments and high oil content. According to recent research, microalgae are currently considered to be a most promising source of high added-value bioproducts such as carbohydrates, vitamins, pigments, polyphenols, flavonoids, lipids and preservative protein-derived compounds [69,70,71].

On the basis that the European Council regulation has considered algae as a food or food ingredient, their use in food technology in general should not constitute any hazard to health [72]. Therefore, their employment in human health has increased in the last decades, including their role as a source of preserving compounds.

### 4.2. Research Carried out on the Preservative Effect of Alga-Derived Compounds as Packing Medium

Ortiz et al. [73] carried out a comparative study of four different seaweed (cochayuyo, frond of *Durvillaea antartica*; sea lettuce, *Ulva lactuca*; ulte, basal part of *D. antartica*; red luche, *Pyropia columbina*) extracts employed as packing media in canned Atlantic salmon (*S. salar*). The presence of alga extracts in the packing media led in all cases to a lower formation of secondary lipid oxidation compounds, which was accompanied by a higher retention of astaxanthin and PUFA. Additionally, the sensory analysis detected a lower oxidised odour development in all alga-treated canned muscle.

**Figure 1:**
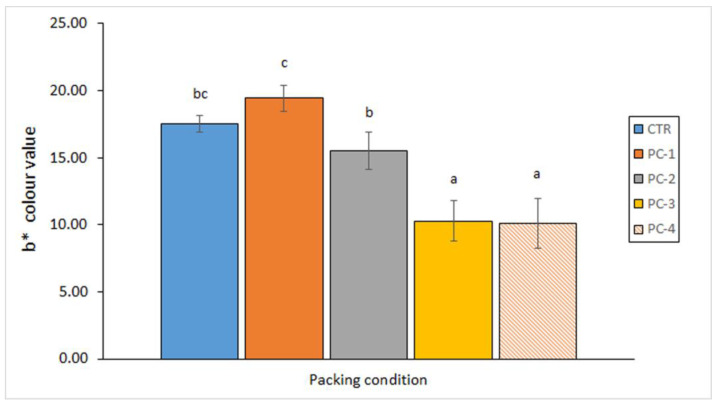
Determination of the b* colour value in canned mackerel (*S. scombrus*) packed under different conditions. Average values accompanied by different letters denote significant differences (*p* < 0.05). Sample abbreviations: CTR, PC-1, PC-2, PC-3 and PC-4 correspond to packing conditions including 0.000, 0.125, 0.250, 0.625 and 1.250 g of extracted alga in the packing medium, respectively. Adapted from Barbosa et al. [74].

The effect on quality changes of including an aqueous *Bifurcaria bifurcata* extract in the packing medium used during Atlantic mackerel (*S. scombrus*) canning was analysed by Barbosa et al. [74]. An inhibitory effect on the lipid oxidation development (fluorescent compound formation) and on the L* colour value increase was observed as a result of the alga addition to the packing medium. Concerning the b* colour parameter, an increasing presence of *B. bifurcata* extract in the packing medium led to a lower value, so that a remarkable decrease was detected by employing the two most concentrated conditions tested (Figure 1). On the contrary, the presence of the alga extract did not produce any effect on the lipid hydrolysis (free fatty acid) development and on the volatile compound (total and trimethylamine) formation.

**Figure 2:**
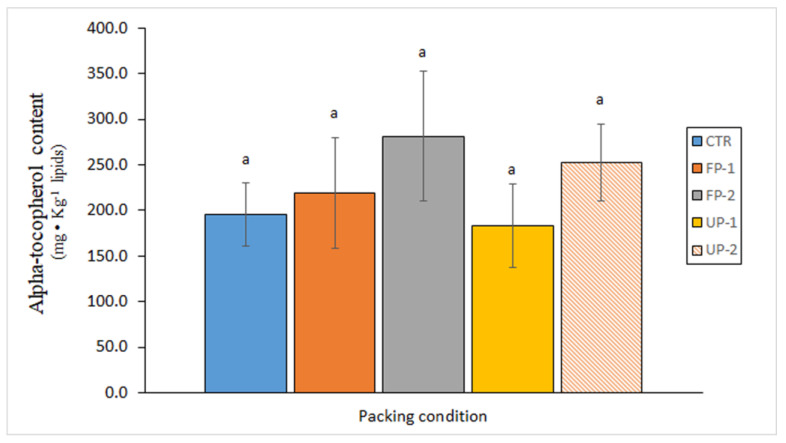
Determination of the alpha-tocopherol content in canned mackerel (*S. colias*) packed under different conditions. No significant differences (*p* > 0.05) were detected (same “a” value for all samples) as a result of the packing medium employed. Sample abbreviations: CTR (Control), FP-1 and FP-2 (low- and high-concentrated *F. spiralis* extract), and UP-1 and UP-2 (low- and high-concentrated *U. lactuca* extract). Adapted from Barbosa et al. [75].

The effect of the aqueous extracts of two algae (*Fucus spiralis* and *U. lactuca*) on the lipid changes in commercial canned Chub mackerel (*Scomber colias*) was analysed by Barbosa et al. [75]. The content of different kinds of lipid classes (phospholipids and sterols) showed marked losses as a result of canning; however, this loss was mostly inhibited by the presence of both algae extracts in the packing medium. Concerning the alpha-tocopherol content in canned muscle, significant (*p* > 0.05) differences were not found by including the algae extracts in the packing media (Figure 2); however, higher average values were observed in the canned fish packed using the highest concentrations of both algae). The oxidation development (formation of fluorescent compounds) showed a marked increase with the canning process; however, this increase was partly inhibited by the addition of antioxidant compounds present in the algae extracts. The oxidation inhibition was found to be more important in the canned fish corresponding to *F. spiralis* packing than in their counterparts corresponding to the *U. lactuca* treatment.

The impact of the addition of different microalgae (*Chlorella minutissima, Isochrysis galbana* and *Picochlorum* sp.) at concentrations of 0.5, 1 and 1.5% *w*/*v* on the texture and sensory attributes of sunflower oil-canned fish burgers was investigated by Atitallah et al. [76]. Compared to the controls, the fish burgers containing the mentioned microalgae revealed better texture and sensory properties. These microalgae-supplemented burgers showed a higher swelling ability as well as higher water- and oil-holding capacities, due to the dietary fibre content of microalgae. Moreover, the microalgae-supplemented burgers were characterised as having low a* and b* colour values, which made the colour appear to be pale orange. The antioxidant activity observed for the microalgae in the canned fish burgers was explained by the presence of pigments (chlorophylls, carotenoids and phycocyanins).

Vieira et al. [77] developed new canned chub mackerel *(S. colias*) products incorporating different edible seaweeds (*Ascophyllum nodosum, F. spiralis, Saccorhiza polyschides, Chondrus crispus, Porphyra* sp. and *Ulva* sp.) in the packing medium. The results showed that the canned chub mackerel incorporating *C. crispus* and *F. spiralis* provided a higher sensory acceptability than any other seaweed-treated canned samples. When compared to the control, all algae-treated samples provided higher levels of different valuable elements such as Cl, Co, Cu, Fe, I, Li, Mg, Mn, Mo, Na, Rb, Se and Sr.

The effect on canned mackerel (*S. colias*) quality of a prior chilling period (up to 9 days) and the presence of an aqueous alga (*F. spiralis*) extract in the packing medium was investigated [78]. A substantial increase in the free fatty acid content was observed in the canned fish by increasing the chilling time; however, the alga extract presence in the packaging medium led to decreased average values. Concerning lipid oxidation development, an increased chilling time led to higher values of the TBARS index and the fluorescent compound formation in the canned mackerel. Remarkably, an increased presence of the alga extract led to a higher peroxide retention and a lower fluorescent compound content. The average colour L* and a* values showed a decrease and an increase, respectively, with previous chilling time; however, such changes were minimised by increasing the alga extract presence in the packing system.

Phenolic extracts from different red seaweeds (*Gracilaria chilensis, Gelidium chilense, Iridaea larga, Gigartina chamissoi, Gigartina skottsbergii* and *Gigartina radula*) were prepared with an ethanol/water mixture (1:1) and added to salmon paste that was subsequently heat treated (90 ± 5 °C for 30 min) [79]. The analysis of all seaweed extracts provided valuable levels of total polyphenols, total flavonoids, as well as antioxidant (DPPH and FRAP assays) and antibacterial power. As a result, all salmon products exhibited a low production of primary lipid oxidation products and a marked protection of essential components (i.e., EPA and DHA) and endogenous antioxidants such as tocopherols and astaxanthin, particularly in the case of *G. skottsbergii, G. chamissoi, G. radula* and *G. chilensis*.

## 5. Effect of Seafood By-Product Packing on the Thermal Stability of Canned Seafood

### 5.1. General Aspects of Preserving Properties of Seafood By-Product Compounds

The technological treatment of marine species gives rise to abundant amounts of by-products. Remarkably, only 50 to 60% of the total catch is used for direct human consumption, so that seafood processing is considered as one of the main sources of by-products (heads, blood, viscera, skin, tails, etc.) among different kinds of food [80,81]. Different kinds and quantities of by-products are generated at the different technological steps between the capture time and final consumption of seafood. The anatomical features of each species and the harvesting and processing methods applied can determine the kinds of by-products that can be obtained.

As a result of this marked formation of undesired products, seafood processing constitutes an important source of environmental contamination unless efforts for their recovery are carried out and their commercial value can be enhanced via the extraction of valuable constituents [82,83]. Therefore, the utilisation of by-products can be considered as an important opportunity for the seafood industry because it can potentially lead to additional incomes as well reduction in the disposal costs of these materials. The valorisation of discarded by-products in the seafood processing industry is among the most important topics discussed in recent years because marine by-products have been reported to contain the same valuable components as the corresponding edible tissues [84,85].

The traditional utilisation of by-products has been as fish meal and oil, fish silage production and small quantities of pet food, fishing bait and fertiliser production. Recent studies have reported that many bioactive components could be incorporated into nutraceutical, functional food formulation or pharmaceutical applications. Remarkably, the highest concentration of high added-value compounds such as minerals, lipids, enzymes, pigments, collagen, vitamins, amino acids, polysaccharides and proteins is often present in the body parts of marine organisms that are commonly discarded [86,87]. As a consequence, the current research carried out by food technologists is addressing great attention in converting seafood by-products into sources of bioactive compounds that could be employed in seafood processing and food processing in general as preservative substances and in human nutrition [88,89].

### 5.2. Research Carried out on the Preservative Effect of Seafood By-Product Compounds as Packing Medium

Lapis et al. [90] investigated the beneficial effect of adding salmon oil in different proportions to canned pink salmon (*O. gorbusha*) produced from fish individuals exhibiting two opposite degrees of skin watermarking (i.e., bright and dark). It was concluded that canned bright pink salmon should be supplemented with at least 1% salmon oil, while supplementation with 2% salmon oil would guarantee a satisfactory ω3 fatty acid level (i.e., EPA and DHA values). Furthermore, a 1–2% addition of salmon oil was not found to be detrimental to the sensory properties. On the other hand, the addition of 4% salmon oil to canned dark pink salmon was found to be detrimental to the texture and taste, while supplementation with 2% salmon oil did not negatively affect the sensory properties of the product. It was concluded that canned dark pink salmon should be supplemented with 2% salmon oil to ensure a valuable ω3 fatty acid content.

Ali et al. [91] formulated a canned tilapia fish luncheon with 20, 25, 30 and 35% of beef fat and stored the resulting products at room temperature (25–35 °C). After a 6-month storage, higher crude fat and energy products were obtained compared to the control fish luncheon, but with lower moisture, crude protein and total ash contents. An increased concentration in the beef fat added led to lower total volatile base nitrogen, trimethylamine and redness (a*) values of the canned tilapia fish luncheon. However, the TBARS value, lightness (L*), yellowness (b*) and hue angle had an opposite trend. The sensory evaluation led to rating scores that ranged between like moderately and like very much for all of the sensory descriptors (7.30–7.93 out of a 10-point score).

After previous in vitro studies of the antioxidant, antihypertensive and anti-inflammatory properties of protein hydrolysates in two sea cucumber species (*Parastichopus tremulus*, *Holothuria forskali*), García et al. [92] developed novel functional food products from *H. forskali* hydrolysates for incorporation in canned mackerel pâté. The new product was shown to be a valuable source of essential elements (i.e., Se and I), total polyphenols and other antioxidants. No differences in odour, colour, texture, flavour and general aspect were found among the mackerel canned pâtés corresponding to the control and hydrolysate-incorporated products.

Canned yellowfin tuna (*T. albacares*) was prepared in addition to a mixture of brine and hydrosol molecules obtained from by-products resulting from the processing of several aromatic plants (i.e., oregano, laurel, sage and lemon balm) that were included in the packing medium [93]. Thirteen antioxidant molecules were detected in the canned tuna fillets, including phenolic acids (gallic acid, vanillic acid, syringic acid and rosmarinic acid), flavonoids (catechin, epicatechin, vanillin, myricetin, rutin, quercetin, luteolin and apigenin) and one hydroxybenzaldehyde (syringaldehyde). Such preservative compounds ranged between 8.86 mg (quercetin) and 512 mg (rosmarinic acid) per 100 g of tuna flesh, thus proving that the addition of hydrosol compound to the packing medium could lead to an enhanced rancidity stability of the tuna fillets during the canning process. The use of hydrosols to preserve canned fillets was presented as a simple, low-cost and effective way to fortify seafood (or any other food product) using by-products of the essential oil industry.

The preservative properties of red alga *Gelidium* sp. flour were studied in a model system consisting of minced mackerel (*S. scombrus*) muscle that was subjected to heating treatment (50 °C for 11 days) [94]. The presence of the aqueous flour extract led to a preserving effect on conjugated diene and triene and free fatty acid formation, so that higher levels of such compounds were detected in the flour-treated samples. In contrast, the addition of alga flour led to a lower lipid oxidation development (i.e., fluorescent compound formation). All effects were found to be more significant after increasing the alga flour concentration and heating time (up to 11 days).

**Figure 3:**
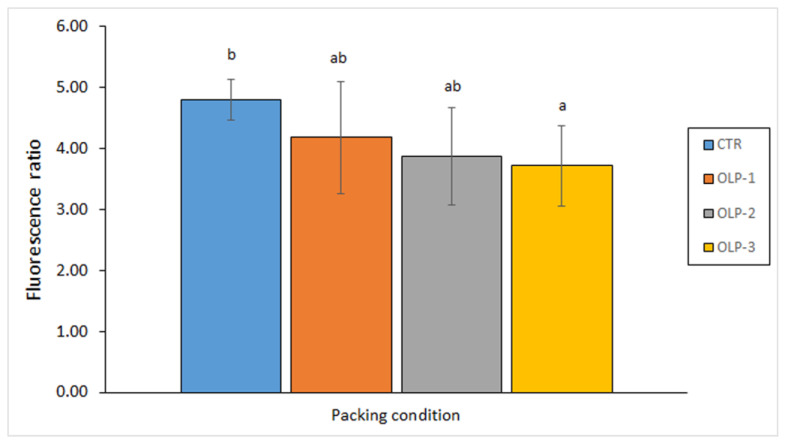
Determination of the fluorescence ratio in canned mackerel (*S. colias*) packed under different packing conditions. Different letters denote significant differences (*p* < 0.05) as a result of the packing medium. Sample abbreviations: CTR (Control), and OLP-1, OLP-2 and OLP-3 (low-, medium- and high-concentrated octopus cooking liquor, respectively). Adapted from Malga et al. [95].

The preservative properties of waste liquor obtained from octopus (*Octopus vulgaris*) cooking during its commercial processing were investigated by Malga et al. [95]. In this study, different concentrations of octopus cooking liquor (OCL) were included in the aqueous packing medium employed for mackerel (*S. colias*) canning. As a result, the presence in the packing medium of the OCL led to a lower TBARS formation. Concerning the formation of tertiary lipid oxidation compounds (i.e., fluorescence ratio) (Figure 3), an increase in the OCL content in the packed medium led to a decreased formation in this kind of oxidation compound. Furthermore, an increasing OCL presence led to an average decrease in the peroxide and free fatty acid content, and to an average increase in the polyene index.

**Figure 4:**
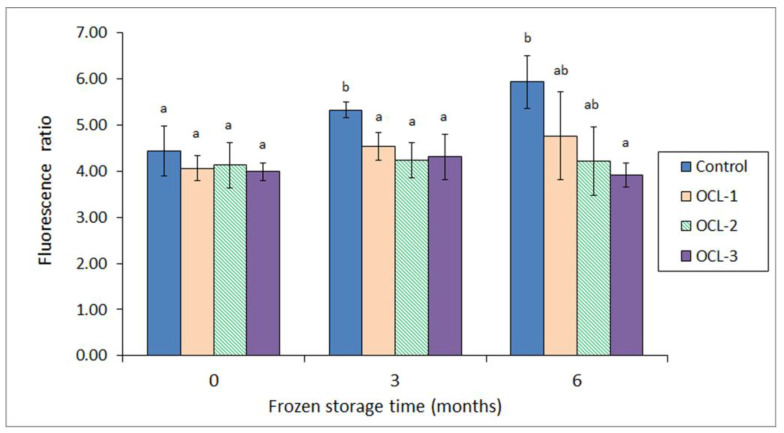
Fluorescent compound formation (i.e., fluorescence ratio) in canned jack mackerel previously subjected to different frozen storage times (0, 3 and 6 months). For each frozen time, different lowercase letters denote significant differences (*p* < 0.05) as a result of the packing medium employed. Packing media: Control (water), OCL-1, OCL-2 and OCL-3 (low-, medium-, and high-concentrated octopus cooking liquor, respectively). Adapted from Méndez et al. [96].

In a later study [96], the same group analysed the effect of the OCL employed as a packing medium in canned horse mackerel (*Trachurus trachurus*) that was previously subjected to frozen storage (3 and 6 months at −18 °C) period. An increased previous storage period led to increased lipid damage. However, the presence of the OCL in the packing medium led to an inhibitory effect on the fluorescent compound formation (Figure 4) as well as to a retention of the phospholipid and free fatty acid values. Additionally, the colour determination revealed lower L* and b* values in the canned fish as a result of the presence of the OCL in the packing medium.

## 6. Final Considerations and Future Research Scopes

In agreement with the research carried out on the thermal stability of seafood during the canning process, the addition of preservative compounds obtained from natural sources to the packing medium can be considered as a valuable strategy to obtain safe, highly-nutritional and attractive processed products. In order to increasing the practical and commercial employment of this preserving strategy, several aspects should be taken into account and on-going research is expected to be extended in next years to offer the consumer an optimised canned seafood. With this basic aim in mind, the following recommendations can be outlined:

### 6.1. Selection of the Kind of Preservative Compounds or Extracts to Be Added

The kind of natural extract (hydrophilic/lipophilic) to be included in the packing medium should be optimised in each case to enhance the sensory and nutritional values of the corresponding canned product, rather than extrapolating the findings made with any other natural source of preserving compounds. For this purpose, knowledge of the preservative extract concentration to be employed would be mandatory. Before practical and commercial development, the preservative compounds to be added to the packing medium should comply with international regulations concerning health risks.

### 6.2. Search for Synergistic Effects among Different Natural Preservatives

It is commonly accepted that the combination of preservatives is one of the most promising strategies for the prevention of processing damage to seafood and food in general. The search for synergistic combinations among different and complementary natural sources (i.e., marine and non-marine) should be intensified to increase the degree of quality of the resulting canned seafood.

### 6.3. Optimisation of Processing Conditions for Each Kind of Product

On the basis of previous studies, a different response can be expected to be produced according to the kind of species (size, fat content, general composition, wild or cultivated, etc.), presentation of the product (whole, fillet, mince, etc.), or other biological aspects of individuals (maturity, sex, eating state, capture season, marine or freshwater species, etc.). Canning conditions should be optimised in each case to enhance the sensory and nutritional values, rather than extrapolating the findings made from any other marine products. Kinetic models including heterogeneous systems may be checked to predict the quality loss and the preservation effectiveness (i.e., antioxidant) in different kinds of marine species.

### 6.4. Detailed Analysis of Chemical Changes

On the basis of the great transformation from the original species to the finished product, the chemical changes occurring in canned seafood would have a decisive effect on the quality loss. In order to obtain a better understanding of such changes, the combination of traditional and advanced analytical tools would be mandatory. Among the advanced tools, the employment of mass spectrometry, nuclear magnetic resonance, electron spin resonance or Raman resonance spectroscopy would provide great advantages to assess the damage mechanisms of the different marine constituents during canning, especially in the case of the lipid fraction. The possible loss of water-soluble vitamins and microelements as a result of a decrease in the water-holding capacity of proteins should be considered. This effect could be especially important in the case of employing hydrophilic packing media.

### 6.5. Consumer’s Requirements

The current and future preparations of seafood should focus on the development of attractive products that fulfil the consumer’s expectations for odour, colour, taste, flavour and general appearance. The presence of preservative compounds in the packing medium should be optimised so that the sensory characteristics that are expected to be present in the final canned seafood are not modified. Innovative and persuasive communication strategies to raise consumer awareness of quality and healthy effects of canned seafood including natural preservative packing would be required.

## Figures and Tables

**Table 1:** Fatty acid ratios (PUFA/STFA and ω3/ω6) in the lipid fraction of initial and canned mackerel muscle.

Packing Medium	Fatty Acid Ratio			
	PUFA/STFA		ω3/ω6	
	Initial	Canned	Initial	Canned
Water	0.90 A (0.10)	1.25 Ba (0.07)	9.04 A (1.10)	10.83 Bb (0.46)
Brine	0.90 A (0.10)	1.26 Ba (0.07)	9.04 A (1.10)	10.75 Bb (0.40)
Sunflower oil	0.90 A (0.10)	1.37 Ba (0.14)	9.04 A (1.10)	8.24 Aa (1.03)
Refined olive oil	0.90 A (0.10)	1.31 Ba (0.09)	9.04 A (1.10)	9.85 Aab (0.84)
Virgin olive oil	0.90 A (0.10)	1.30 Ba (0.06)	9.04 A (1.10)	9.95 Aab (0.76)

* For each ratio, different capital letters denote significant differences (*p* < 0.05) as a result of canning; different lowercase letters denote significant differences (*p* < 0.05) as a result of the packing medium. Adapted from Prego et al. [39].
